# Correction: DNA Damage, Homology-Directed Repair, and DNA Methylation

**DOI:** 10.1371/journal.pgen.1006605

**Published:** 2017-02-10

**Authors:** Concetta Cuozzo, Antonio Porcellini, Tiziana Angrisano, Annalisa Morano, Bongyong Lee, Alba Di Pardo, Samantha Messina, Rodolfo Iuliano, Alfredo Fusco, Maria R. Santillo, Mark T. Muller, Lorenzo Chiariotti, Max E. Gottesman, Enrico V. Avvedimento

Fig 5B shows a bivariate analysis of GFP fluorescence (FL1) vs Side Scatter (SSC). The FL2 axis labels in Fig 5B are incorrect, as they represent the SSC and not fluorescence. The authors have provided a corrected Fig 5 and legend here.

**Fig 5:**
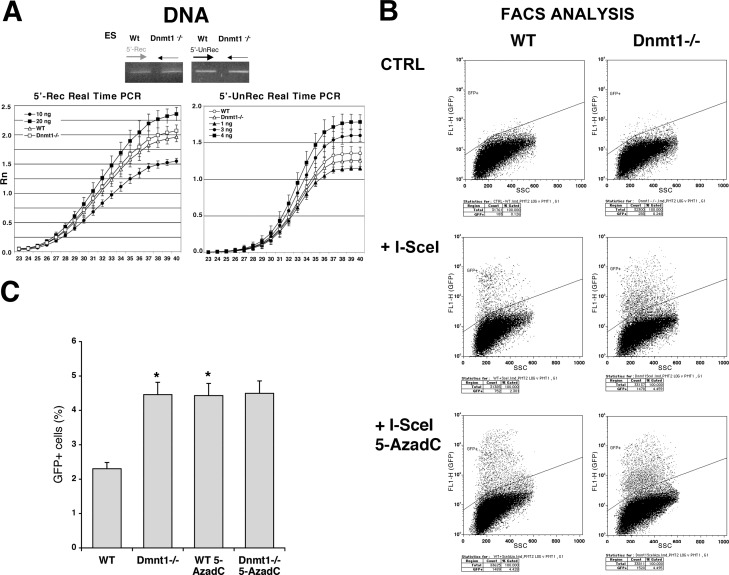
DNA methyl transferase I inhibits the expression of recombinant GFP genes. Wild type or Dnmt1-/- ES cells carrying DR-GFP were transfected with the I-SceI expression vector and PSVbGal, grown 4 days and analyzed for GFP recombination and expression. **A.** Genomic DNA from the two cell lines was PCR-amplified with non-recombinant (5’-UnRec) and recombinant (5’-Rec) primers. The specificity of the products and the linearity of the reactions were controlled as described in Materials and Methods. RealTime-PCR of the same samples carried out as described in Materials and Methods. **B.** Bivariate FACS analysis of cells transfected with I-SceI (GFP vs Side Scatter, SSC). The gating of GFP^+^ cells was created to exclude up the 99.5% of WT, untransfected ES cells. The same gating applied to Dnmt1-/- cells shows a significant increase in the population expressing GFP. Following I-SceI transfection, Dnmt1-/- cells were treated with 5-AzadC as described in Materials and Methods. Treatment with 5-AzadC increased the fraction of cells expressing GFP in wild type ES but did not enhance the expression of GFP in the Dnmt1-/- cells **C.** histogram showing the fraction of GFP^+^ cells derived from 3 experiments. To get reliable values of differential GFP fluorescence in ES and Dnmt1-/- cells, we compared the percentage of GFP+ cells, normalized for the transfection efficiency in 6 experiments (3 in duplicate) with the Wilcoxon Kruskal-Wallis Test, * p < 0.012 versus wild type.
